# Platelet RNA signature independently predicts ovarian cancer prognosis by deep learning neural network model

**DOI:** 10.1093/procel/pwac053

**Published:** 2022-11-07

**Authors:** Chun-Jie Liu, Hua-Yi Li, Yue Gao, Gui-Yan Xie, Jian-Hua Chi, Gui-Ling Li, Shao-Qing Zeng, Xiao-Ming Xiong, Jia-Hao Liu, Lin-Li Shi, Xiong Li, Xiao-Dong Cheng, Kun Song, Ding Ma, An-Yuan Guo, Qing-Lei Gao

**Affiliations:** Department of Gynecological Oncology, Tongji Hospital, Tongji Medical College, Huazhong University of Science and Technology, Wuhan 430030, China; National Clinical Research Center for Obstetrics and Gynecology, Cancer Biology Research Center (Key Laboratory of the Ministry of Education), Tongji Hospital, Tongji Medical College, Huazhong University of Science and Technology, Wuhan 430030, China; Center for Artificial Intelligence Biology, Hubei Bioinformatics & Molecular Imaging Key Laboratory, Key Laboratory of Molecular Biophysics of the Ministry of Education, College of Life Science and Technology, Huazhong University of Science and Technology, Wuhan 430074, China; Department of Gynecological Oncology, Tongji Hospital, Tongji Medical College, Huazhong University of Science and Technology, Wuhan 430030, China; National Clinical Research Center for Obstetrics and Gynecology, Cancer Biology Research Center (Key Laboratory of the Ministry of Education), Tongji Hospital, Tongji Medical College, Huazhong University of Science and Technology, Wuhan 430030, China; Department of Gynecological Oncology, Tongji Hospital, Tongji Medical College, Huazhong University of Science and Technology, Wuhan 430030, China; National Clinical Research Center for Obstetrics and Gynecology, Cancer Biology Research Center (Key Laboratory of the Ministry of Education), Tongji Hospital, Tongji Medical College, Huazhong University of Science and Technology, Wuhan 430030, China; Center for Artificial Intelligence Biology, Hubei Bioinformatics & Molecular Imaging Key Laboratory, Key Laboratory of Molecular Biophysics of the Ministry of Education, College of Life Science and Technology, Huazhong University of Science and Technology, Wuhan 430074, China; Department of Gynecological Oncology, Tongji Hospital, Tongji Medical College, Huazhong University of Science and Technology, Wuhan 430030, China; National Clinical Research Center for Obstetrics and Gynecology, Cancer Biology Research Center (Key Laboratory of the Ministry of Education), Tongji Hospital, Tongji Medical College, Huazhong University of Science and Technology, Wuhan 430030, China; Cancer Center, Union Hospital, Tongji Medical College, Huazhong University of Science and Technology, Wuhan 430022, China; Department of Gynecological Oncology, Tongji Hospital, Tongji Medical College, Huazhong University of Science and Technology, Wuhan 430030, China; National Clinical Research Center for Obstetrics and Gynecology, Cancer Biology Research Center (Key Laboratory of the Ministry of Education), Tongji Hospital, Tongji Medical College, Huazhong University of Science and Technology, Wuhan 430030, China; Department of Gynecological Oncology, Tongji Hospital, Tongji Medical College, Huazhong University of Science and Technology, Wuhan 430030, China; National Clinical Research Center for Obstetrics and Gynecology, Cancer Biology Research Center (Key Laboratory of the Ministry of Education), Tongji Hospital, Tongji Medical College, Huazhong University of Science and Technology, Wuhan 430030, China; Department of Gynecological Oncology, Tongji Hospital, Tongji Medical College, Huazhong University of Science and Technology, Wuhan 430030, China; National Clinical Research Center for Obstetrics and Gynecology, Cancer Biology Research Center (Key Laboratory of the Ministry of Education), Tongji Hospital, Tongji Medical College, Huazhong University of Science and Technology, Wuhan 430030, China; Cancer Center, Union Hospital, Tongji Medical College, Huazhong University of Science and Technology, Wuhan 430022, China; Department of Gynecology and Obstetrics, The Central Hospital of Wuhan, Tongji Medical College, Huazhong University of Science and Technology, Wuhan 430030, China; Department of Gynecological Oncology, Women’s Hospital, School of Medicine, Zhejiang University, Hangzhou 310011, China; Gynecological Oncology Key Laboratory, Qilu Hospital, Shandong University, Jinan 250100, China; Department of Gynecological Oncology, Tongji Hospital, Tongji Medical College, Huazhong University of Science and Technology, Wuhan 430030, China; National Clinical Research Center for Obstetrics and Gynecology, Cancer Biology Research Center (Key Laboratory of the Ministry of Education), Tongji Hospital, Tongji Medical College, Huazhong University of Science and Technology, Wuhan 430030, China; Center for Artificial Intelligence Biology, Hubei Bioinformatics & Molecular Imaging Key Laboratory, Key Laboratory of Molecular Biophysics of the Ministry of Education, College of Life Science and Technology, Huazhong University of Science and Technology, Wuhan 430074, China; Department of Gynecological Oncology, Tongji Hospital, Tongji Medical College, Huazhong University of Science and Technology, Wuhan 430030, China; National Clinical Research Center for Obstetrics and Gynecology, Cancer Biology Research Center (Key Laboratory of the Ministry of Education), Tongji Hospital, Tongji Medical College, Huazhong University of Science and Technology, Wuhan 430030, China

**Dear Editor,**

Platelets are circulating anucleate cytoplasmic fragments of megakaryocytes and characterized by their functions in wound healing and vascular integrity maintenance. Increasing evidence highlights the extensive reciprocal signaling interactions between platelets and tumor cells (Haemmerle et al., 2018). Tumor cells activate and aggregate platelets to sustain proliferation (Cho et al., 2012), resist apoptosis, and promote metastasis (Haemmerle et al., 2017). Besides paraneoplastic thrombocytosis (Stone et al., 2012), platelets undergo morphological changes in the microtubules, mitochondria, and storage granules (Wang et al., 2015) and transcriptional reprogramming due to intrinsic alternations of gene expression and the uptake of tumor-derived RNAs through a biological process called education (Roweth and Battinelli, 2021). Tumor-educated platelets (TEPs) have enabled accurate discrimination between patients with cancer and healthy controls (Best et al., 2015; Xu et al., 2022) and emerged as the latest component of liquid biopsy (Haemmerle et al., 2018). Though molecular profiling of platelets makes great strides in disease detection, its capability for mortality risk stratification in patients with cancer remains unclear. Despite the advanced understanding of TEPs as liquid biopsy component, uncertainties remain regarding the versatility of TEPs since circulating tumor cells (CTCs) and circulating tumor DNA (ctDNA) embrace multifaceted utilities beyond cancer detection.

Ovarian cancer is an intractable disease that often evades early diagnosis and defies treatment. Over 75% of diseases have progressed to an advanced stage when initially diagnosed, harboring a 5-year relative survival rate of 29% (Siegel et al., 2022). Ovarian cancer has intricate heterogeneity between and within subtypes and refers to a group of molecularly and etiologically different neoplasms that simply share an anatomical location (Vaughan et al., 2011). Accurate survival prediction tailored to women with ovarian cancer is conducive to classifying risk and estimating survival more objectively in clinical practice, but riddled with challenges. Currently, besides classic prognostic factors featured by TCGA subtype, patient age, disease stage, postoperative residual disease, liquid biopsy components including CTCs, ctDNA, cell-free RNA, and exosomes have been reported to associate with ovarian cancer prognosis (Zhu et al., 2022). However, the capabilities of TEPs to predict cancer outcomes remain enigmatic.

During clinical practice, we observed that ovarian cancer patients with thrombocytosis had increased tumor load and decreased chance for R0 resection (cytoreductive surgery that removed all macroscopic diseases) than those with normal platelet counts. Pretreatment platelet count was significantly higher in patients that failed to achieve R0 resection than in those with R0 status (*P* = 0.0033, Fig. 1A). Consistently, thrombocytosis (>350 × 10^9^/L) was proven to be significantly associated with curtailed ovarian cancer survival (Stone et al., 2012). To ascertain the association in the Chinese population, we collected treatment-naïve blood samples of 2404 patients and retrieved corresponding clinical and follow-up data from China Real-world Gynecological Oncology Platform. Their baseline characteristics are presented in Table S1. The results demonstrated that thrombocytosis (>350 × 10^9^/L) was significantly associated with shortened progression-free survival (PFS, Log-rank *P* = 0.01, Fig. S1) and overall survival (OS, Log-rank *P* < 0.0001, Fig. 1B) of ovarian cancer.

**Figure 1.**
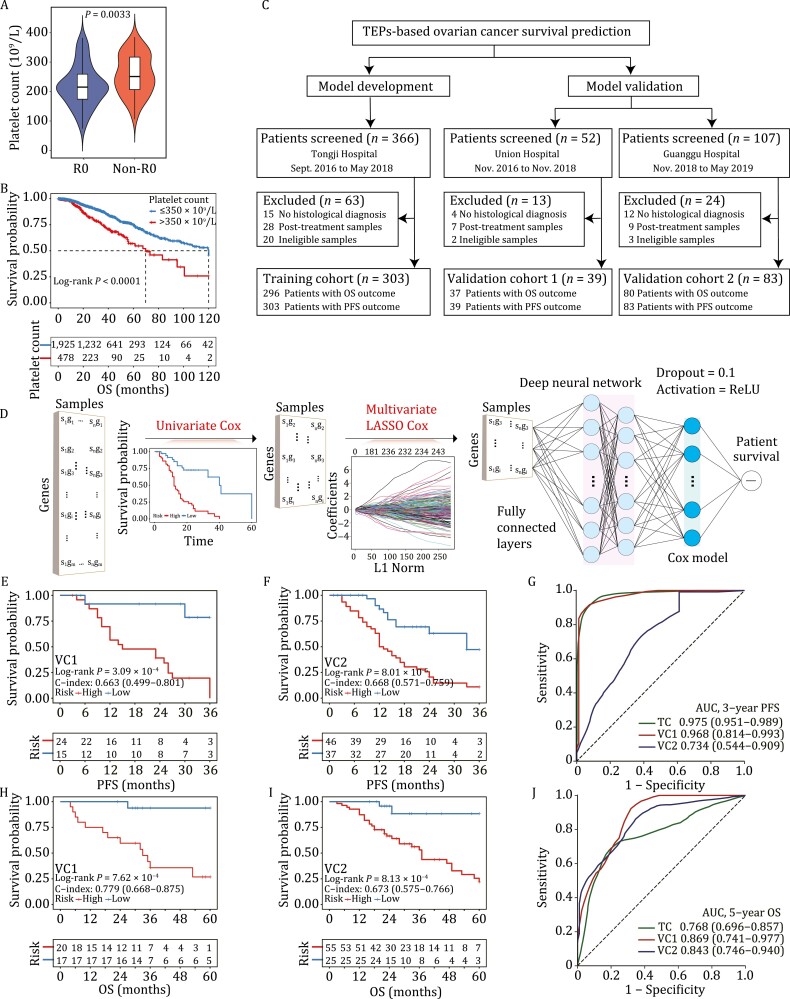
Tumor-educated platelets enable ovarian cancer survival prediction. (A) Comparison of treatment-naïve platelet count between patients that achieved R0 resection (R0, *n* = 57) and patients that did not achieve R0 resection (non-R0, *n* = 74). (B) Kaplan-Meier survival analysis showed that pretreatment thrombocytosis (>350 × 10^9^/L) was significantly associated with shortened overall survival in 2404 patients with ovarian cancer. (C) Participant enrollment. Ineligible samples were those with low quality (RNA integrity number < 7) or quantity (< 10 picogram) of total RNA. (D) Model development. DeepCox combines deep learning neural network with survival models to learn survival-related patterns from platelet RNA profiles. DeepCox consists of univariate Cox regression to filter the nonsurvival-related genes, multivariate LASSO Cox regression to select multivariate contributing genes, fully connected layers that provide additional nonlinear transformations of gene features (The dropout = 0.1 and activation = ReLU), and a Cox proportional hazards layer that models time-to-event data such as overall survival and progression-free survival. (E–J) Model performance. Kaplan-Meier and ROC analyses by DeepCox in two prospective validation cohorts. Kaplan-Meier plots for PFS analysis in VC1 (E) and VC2 (F). Kaplan-Meier plots for OS analysis in VC1 (H) and VC2 (I). ROC analysis of 3-year PFS (G) and 5-year OS (J) in VC1 and VC2. OS, overall survival. PFS, progression-free survival; LASSO, least absolute shrinkage and selection operator; VC, validation cohort; ROC, receiver operating characteristic curve; AUC, area under the ROC curve.

To further investigate the association between the transcriptome of TEPs and ovarian cancer survival, we screened 525 consecutive patients from three hospitals in China between September 2016 and May 2019, excluding 100 patients (patients without histological diagnosis, *n* = 31; posttreatment patients, *n* = 44; patients with ineligible samples, *n* = 25), and performed RNA sequencing of TEPs in 425 participants. Specifically, training cohort, prospective validation cohort 1 (VC1), and prospective validation cohort 2 (VC2) comprised 303, 39, and 83 patients, respectively. The flowchart of participant enrollment is shown in Fig. 1C. Their baseline characteristics are summarized in Table S2. After a median follow-up of 35 months, the median OS and PFS were comparable between training cohort (OS, 37; PFS, 20) and VC1 (OS, 33; PFS, 23), and worsened in VC2 (OS, 25; PFS, 15). The median age (years) in three cohorts was balanced [training cohort, 51, IQR: (45, 59); VC1, 52, IQR: (47, 56); VC2, 52, IQR: (46, 59)]. The majority of participants received platinum-based chemotherapy and responders accounted for 71.5% (*n* = 181), 66.7% (*n* = 18), and 76.1% (*n* = 54) of patients in training cohort, VC1, and VC2, respectively. Both poly (ADP-ribose) polymerase (PARP) inhibitors (total *n* = 9) and angiogenesis inhibitors (total *n* = 43) were used in low frequency among three cohorts. The study design including eligibilities and principles of follow-ups is elaborated in the Supplementary Methods.

Pretreatment blood samples collected from three hospitals were processed using the same standardized protocols (Best et al., 2015). The method for platelet sample processing (Supplementary Methods) ensured platelet purity (Fig. S2) and prevented platelets from activation within 48 hours (Fig. S3). Raw RNA-seq data were subjected to our in-house RNA-seq pipeline. Data normalization and batch effect removal were performed before model development (Supplementary Methods). Fig. 1D shows the DeepCox workflow where univariate Cox proportional hazards regression model, multivariable least absolute shrinkage and selection operator (LASSO) Cox regression model, and deep neuron network architecture were used for gene selection and model development. DeepCox modeling yielded a platelet signature of 100 genes for ovarian cancer survival prediction (Table S3). The signature included several genes (e.g., *RALA*, *MYLK*, *RAP1B*, and *SRC*) that encode proteins that play significant roles in platelet lifeline including proplatelet formation, platelet aggregation, spreading, and activation (Chrzanowska-Wodnicka et al., 2005; Meinders et al., 2015; Wersäll et al., 2018; De Kock and Freson, 2020). To illustrate the biological effects of these genes, we did enrichment analysis based on the 100 genes by gene ontology (Fig. S4A), REACTOME pathway database (Fig. S4B), Pathway Interaction Database (Fig. S4B), and Molecular Signatures Database cancer hallmarks (Fig. S4C), respectively. DeepCox predictions generated two groups with favorable (low risk) or dismal (high risk) outcome by risk scores. Kaplan-Meier survival analysis in training cohort revealed that the signature displayed high concordance index (C-index) for PFS (0.891) and OS (0.713) (Fig. S5A and S5B).

To characterize the generalizability of DeepCox, we tested its efficiency in two prospective validation cohorts. The follow-up data collected in March 2022 were analyzed. High-risk patients had a significantly impaired 3-year PFS than their low-risk counterparts in VC1 [C-index = 0.663, 95% confidence interval (CI) 0.499–0.801, Log-rank *P* = 3.09 × 10^–4^, Fig. 1E] and VC2 (C-index = 0.668, 95% CI 0.571–0.759, Log-rank *P* = 8.01 × 10^–5^, Fig. 1F). The area under the time-dependent receiver operating characteristic (ROC) curve for 3-year PFS was 0.968 (95% CI 0.814–0.993) in VC1 and 0.734 (95% CI 0.544–0.909) in VC2 (Fig. 1G). Similarly, patients with high-risk scores displayed a significantly shortened OS in VC1 (C-index = 0.779, 95% CI 0.668–0.875, Log-rank *P* = 7.62 × 10^–4^, Fig. 1H) and VC2 (C-index = 0.673, 95% CI 0.575–0.766, Log-rank *P* = 8.13 × 10^–4^, Fig. 1I) than those scored lower. For 5-year OS, DeepCox demonstrated an area under the time-dependent ROC curve of 0.869 (95% CI 0.741–0.977) and 0.843 (95% CI 0.746–0.940) in VC1 and VC2, respectively (Fig. 1J).

Risk score distribution estimated by DeepCox is available in Fig. 2A and 2B. The significant association between risk score and ovarian cancer survival was corroborated using univariate Cox proportional hazards regression analysis [PFS: hazard ratio (HR) = 8.51 (95% CI 6.34–11.42), *P* = 3 × 10^–46^, Fig. S6A; OS: HR = 5.5 (95% CI 2.43–12.44), *P* = 4.2 × 10^–5^, Fig. S6B]. Univariate analysis also demonstrated the established prognostically relevant variables including disease stage and tumor residual volume. To understand whether DeepCox predictions were independent of recognized predictive variables, we applied multivariable Cox proportional hazards regression analysis with risk scores and known predictors as covariates (Fig. 2C and 2D). After multivariable analysis for platelet count, patient age, serum cancer antigen 125 (CA125) concentration, residual tumor, and International Federation of Gynecology and Obstetrics (FIGO) stage, higher DeepCox risk score was significantly associated with worsened survival and exhibited the most striking HRs [PFS: HR = 6.83 (95% CI 4.97–9.39), *P* = 1.9 × 10^–32^, Fig. 2C; OS: HR = 4.13 (95% CI 2.79–6.11), *P* = 1.4 × 10^–12^, Fig. 2D], suggesting that DeepCox risk score was an independent predictor of ovarian cancer survival. We plotted nomograms to pictorially present the mathematical formula and facilitate potential clinical applications (Fig. 2E and 2F). Each variable was listed separately, with a corresponding number of points assigned to a particular magnitude of the variable. The cumulative point score for all variables was matched to a scale of outcome including probabilities of 1-year, 2-year, and 3-year PFS or 3-year and 5-year OS.

**Figure 2.**
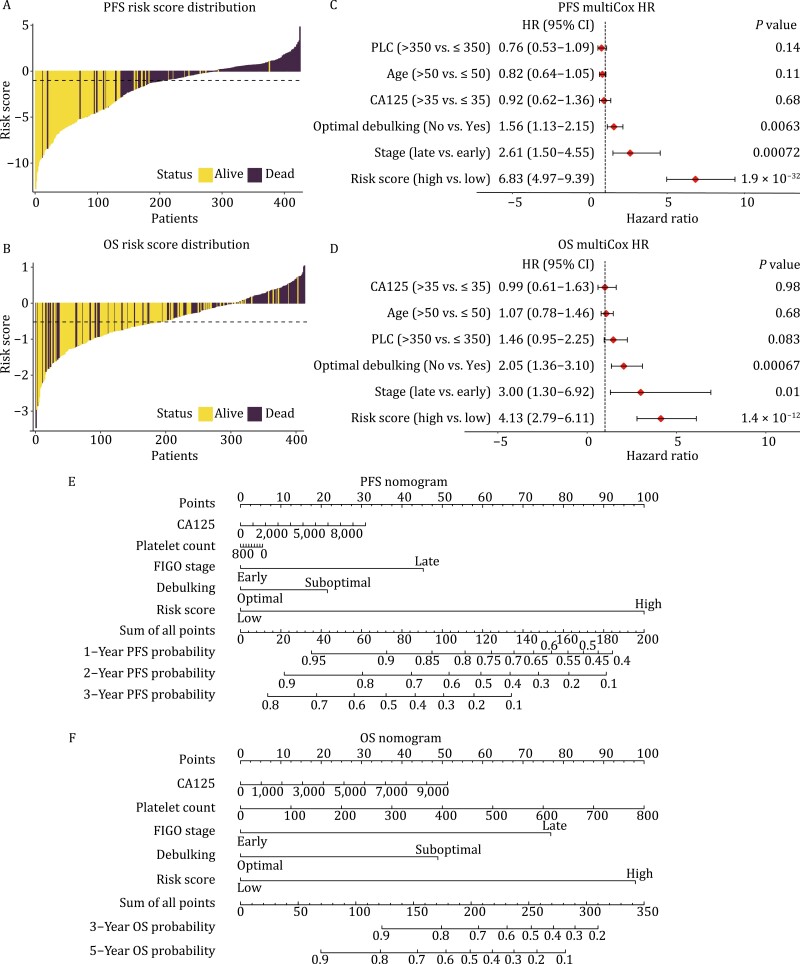
Tumor-educated platelets independently predict ovarian cancer survival. The distribution of risk score estimated by DeepCox for PFS (A) and OS (B). Multivariable Cox regression analysis for PFS (C) and OS (D). Nomograms for 3-year PFS (E) and 5-year OS (F). PLC, platelet count (×10^9^/L). HR, hazard ratio; PFS, progression-free survival; OS, overall survival.

Advanced age and thrombocytosis have clinical significance to portend dismal survival of ovarian cancer. However, univariate and multivariable analyses failed to detect their statistical significance (Figs. 2C, 2D, and S6B). In a larger population (*n* = 2404), similar to thrombocytosis (Figs. 1B and S1), advanced age (>50 years) was proven an unfavorable prognostic predictor for ovarian cancer (both Log-rank *P* < 0.0001, Fig. S7A and S7B). Since the response to chemotherapy determines patient survival in ovarian cancer, we analyzed the differentially expressed genes of platelet transcriptome between responders and nonresponders to chemotherapy (Fig. S8). However, the differentially expressed genes were in a limited number and only shared one gene with the predictive signature.

In conclusion, driven by deep learning algorithm, a platelet RNA signature comprising 100 genes enabled accurate mortality risk stratification of treatment-naïve patients with ovarian cancer. Moreover, TEPs-powered personalized survival prediction showed efficiency and promise in two small-scale prospective validation cohorts of ovarian cancer after a median follow-up of 35 months. Importantly, an escalation of DeepCox risk score was demonstrated as an independent unfavorable predictor for ovarian cancer survival. Together, this study provided scientific evidence for a hypothesis based on bedside observations and discovered a predictive signature with encouraging potential of clinical translation for ovarian cancer survival.

## Supplementary data

The online version contains supplementary material available at https://doi.org/10.1093/procel/pwac053.

pwac053_suppl_Supplementary_Material

pwac053_suppl_Supplementary_Table_S1

pwac053_suppl_Supplementary_Table_S2

pwac053_suppl_Supplementary_Table_S3

## Footnotes

This study was supported by the National Science and Technology Major Sub-Project (2018ZX10301402-002), National Natural Science Foundation of China (81772787, 82072889, 31822030, and 31771458), Technical Innovation Special Project of Hubei Province (2018ACA138), Fundamental Research Funds for the Central Universities (2019kfyXMBZ024), and Wuhan Municipal Health Commission (WX18Q16).

The authors have no conflicts of interest to declare that are relevant to the content of this article.

This study was approved by the institutional review board at Tongji Medical College, Huazhong University of Science and Technology (S253) and performed in compliance with the principles of Declaration of Helsinki. This study was registered in Chinese Clinical Trial Registry as ChiCTR2100046452.

Written informed consent was obtained from all individual participants included in the study.

The datasets generated or analyzed during the current study are available from the corresponding author on reasonable request.

The code used to develop and evaluate the model is available on GitHub.

Conceptualization, AYG, QLG; Data curation, CJL, YG; Formal analysis, CJL, HYL, JHC, XL; Funding acquisition, AYG, QLG; Investigation, CJL, HYL, YG; Methodology, CJL, GYX, XL, AYG; Project administration, AYG, QLG; Resources, GLL, SQZ, XMX, JHL, LLS, XDC, KS; Software, CJL, GYX, AYG; Supervision, AYG, QLG; Validation, all authors; Visualization, CJL, HYL; Writing-original draft, CJL, HYL; Writing-review & editing, all authors.
