# Supplementary material for: Platelet RNA signature independently predicts ovarian cancer prognosis by deep learning neural network model
Source: Protein Cell. 2022 Nov 7;14(8):618–22. doi: 10.1093/procel/pwac053 (PMC10392027; doi:10.1093/procel/pwac053)
Supplement: pwac053_suppl_Supplementary_Material [file pwac053_suppl_supplementary_material.pdf]

Supplementary material to "Platelet RNA signature independently predicts ovarian cancer prognosis by deep learning neural network model"

Chun-Jie Liu[1,2,3,#], Hua-Yi Li[1,2,#], Yue Gao[1,2,#], Gui-Yan Xie[3], Jian-Hua Chi[1,2], Gui-Ling Li[4], Shao-Qing Zeng[1,2], Xiao-Ming Xiong[1,2], Jia-Hao Liu[1,2], Lin-Li Shi[4], Xiong Li[5], Xiao-Dong Cheng[6], Kun Song[7], Ding Ma[1,2], An-Yuan Guo[3*] and Qing-Lei Gao[1,2,*]

Supplementary Methods

Study design

We included consecutive patients with a clinical diagnosis of ovarian cancer in the Department of Gynecologic Oncology of the three hospitals and excluded patients without histological diagnosis, post-treatment patients, and patients with low quality (integrity number < 7) or quantity (< 10 picogram) of total RNA. All eligible participants were histologically confirmed based upon 2014 World Health Organization Classification for Ovarian Tumors (Kurman et al., 2014). FIGO stages I and IIA were classified as early-stage, whereas FIGO stages IIB to IV malignancies were considered late-stage (Prat, 2014). Clinical data related to patient age, histology, FIGO stage, and residual tumor volume after primary debulking surgery were retrieved from electronic health records and proofread by two investigators (YG and SQZ) independently. Serum CA125 measurements and platelet-associated laboratory tests were performed within one week before treatment against cancer initiated. Participants were treated as considered appropriate by the gynecologic oncologists in accordance with the National Comprehensive Cancer Network clinical practice guidelines for ovarian cancer (https://www.nccn.org). Follow-ups were supervised and documented by specialized persons. The last follow-up was conducted in Mar. 2022. In the intervening years, physical examination and CA125 measurements were assessed every 3 months for the first 2 years and twice per year thereafter. Chest radiography and abdominopelvic computed tomography scan or magnetic resonance imaging were performed as clinically indicated. The co-endpoints of interest were 3-year PFS, according to RECIST v1.1, and 5-year OS. PFS was defined as the time from diagnosis to progression, recurrence, or loss to follow-up, and OS was the interval from diagnosis to death or last follow-up. This study was reported following Transparent Reporting of

a multivariable prediction model for Individual Prognosis or Diagnosis guidelines (Collins et al., 2015).

Processing of blood samples, platelet isolation, and RNA extraction

Peripheral venous blood was stored in 10 mL purple-cap BD vacutainers containing the EDTA anticoagulant and processed within 48 hours. Platelets were isolated via a standard gradient centrifugation method (Best et al., 2019). Platelet pellets were gently resuspended in RNAlater (Thermo Scientific) and incubated at 4℃ overnight and transferred to –80℃ after sharply freezing overnight in liquid nitrogen for future use. Platelet isolation and RNA extraction for samples derived from three Chinese hospitals were conducted using the same protocol proposed by Best et al (Best et al., 2017; Best et al., 2019). For samples with total RNA ≥ 50 nanogram, total RNA was extracted from the platelets using Trizol (Invitrogen, Carlsbad, CA, USA) in accordance with the manufacturer's instructions. The mix was centrifuged at 12 000 × *g* for 5 min at 4℃. The supernatant was transferred into a new Eppendorf tube with 0·3 mL chloroform/isoamyl alcohol (24:1). The mix was shaken vigorously for 15 s and then centrifuged at 12 000 × *g* for 10 min at 4℃. The upper aqueous phase containing RNA was transferred into a new tube with an equal volume of isopropyl alcohol and centrifuged at 12 000 × *g* for 20 min at 4℃. After discarding the supernatant, the RNA pellet was washed twice with 1 mL 75% ethanol, and the mix was centrifuged at 12 000 × *g* for 3 min at 4℃ to collect residual ethanol, followed by air-drying of the pellet for 5–10 min in the biosafety cabinet. Finally, 25~100 μL of DEPC-treated water was added to dissolve the RNA pellet. Subsequently, total RNA was qualified and quantified using a Nano Drop spectrophotometer and an Agilent 2100 bioanalyzer (Thermo Fisher Scientific, MA, USA).

For samples with total RNA < 50 nanogram, total RNA was extracted from platelets using the RNeasy Micro Kit (QIAGEN, GER) in accordance with the manufacturer's instructions. Appropriate platelets were ground to powder with liquid nitrogen and then transferred into a new tube with an appropriate volume of Buffer RL and 1 volume 70% ethanol. The mixture was transferred into a RNeasy MinElute spin column and centrifuged at ≥ 8000 × *g* for 15 s. After discarding the flow-through, Buffer RW1, DNase I, Buffer RPE, and 80% ethanol were added and then sequentially centrifuged. The RNeasy MinElute spin column containing RNA was placed in a new 2-mL collection tube and centrifuged with lid opened at 12 000 × *g* for 5 min to

dry the membrane and then transferred to a new 1 5-mL tube with 14 μL RNase-free water. Finally, the tubes were centrifuged for 1 min at 12 000 × *g* to elute the RNA. Total RNA was qualified and quantified using a Nano Drop and Agilent 2100 bioanalyzer (Thermo Fisher Scientific, MA, USA).

For samples with total RNA > 50 nanogram, oligo (dT)-attached magnetic beads were used to purify mRNA. Purified mRNA was fragmented with fragment buffer at 94℃ for 5min. Thereafter, the first strand of cDNA was generated using the First Strand reaction system via PCR and then the second strand of cDNA was generated. The reaction product was purified using Ampure XP Beads and dissolved in EB solution. The quality and quantity of the library were assessed via two methods to ensure the high quality of the sequencing data: one method involved assessing the distribution of the fragment sizes using the Agilent 2100 bioanalyzer; the other method involved quantifying the library via real-time quantitative PCR. The qualified library was amplified on cBot to generate the cluster on the flowcell. Moreover, the amplified flowcell will be sequenced single-end on the HiSeq4000 or HiSeq X-ten platform (BGI-Shenzhen, China).

For samples with total RNA between 10 picogram and 50 nanogram, the platelet RNA was amplified with oligo-dT and dNTPs, incubated at 72℃, and immediately placed on ice, followed by reverse transcription to form cDNA, based on the polyA tail method. The template was switched to the 5′ end of the RNA, and full-length cDNA was generated via PCR. The Agilent 2100 bioanalyzer instrument (Agilent High Sensitivity DNA Reagents) was used to determine the average molecule length of the PCR product. The cDNA library was quantified using the Qubit dsDNA HS Assay Kit (Thermo Fisher Scientific, Waltham, MA, USA) for accurate quantification, followed by fragmentation with fragment buffer. Thereafter, the A-Tailing Mix and RNA Index Adapters were added for end repair. The cDNA fragments with adapters were amplified via PCR. The PCR products were purified using Ampure XP Beads and then were size-selected. The final library was quantitated using two methods to ensure the high quality of the sequencing data: one method involved determining the average molecule length by using the Agilent 2100 bioanalyzer instrument (Agilent DNA 12000 Reagents); the other method involved quantifying the library via real-time quantitative PCR (qPCR). The qualified libraries were amplified using cBot to generate the cluster on the flowcell. The amplified flowcell was sequenced single-end on the HiSeq4000 platform (BGI-Shenzhen, China).

Processing of raw RNA-seq data

Raw RNA-seq data in FASTQ format files were subjected to our in-house RNA-seq pipeline (https://github.com/chunjie-sam-liu/Deeplatelet). Briefly, raw reads were trimmed by FastQC v.0.11.8 and clean reads were mapped to human reference genome (GRCh37) by STAR v2.7.0 (Dobin et al., 2013). To quantify gene expression, aligned reads were subject to HTSeq v0.11.1 with Ensembl gene annotation version 87 (Anders et al., 2015; Yates et al., 2020). Samples with total read count less than 5×10^6 were excluded. Genes with <10 reads in 10% of the cohort samples were excluded.

Data normalization and batch effect removal

Raw read counts of samples in training cohort were normalized and dispersion was estimated by R-package DESeq2 (Love et al., 2014). The yielded dispersion values were assigned as the dispersion of validation cohorts and the same method was applied to data normalization in validation cohorts. To exclude samples with low inter-sample correlation, we used "Bigcor" function of R-package propagate v1.0.6 to perform Pearson correlation, which excluded one sample with a correlation coefficient less than 0.4 from training cohort. To minimize the influences of library size and known batches for modeling and prediction, we investigated potential confounding factors with surrogate variables identified via R-package svaseq with default parameters (Leek, 2014). Each estimated surrogate variable was correlated with the potential confounding factors. The continuous variables were correlated to surrogate variables by Pearson correlation and categorical variables were compared using a two-sided Student's *t*-test. These identified confounding factors were used to adjust the normalized data by limma (Ritchie et al., 2015).

DeepCox model development

For time-to-event prediction, we devised a DeepCox workflow by selecting prognostic genes and combining deep neuron network to build deep Cox regression model (Katzman et al., 2018; Nagpal et al., 2021). We subjected normalized data in training cohort to univariate Cox proportional hazards regression model by R-package survival v3.2.7. Genes with Cox *P* value less than 0.05 were reserved for multivariate LASSO Cox regression. 10-fold cross validation multivariate LASSO Cox proportional hazards regression was performed to optimize minimum

lambda, which was used to extract model coefficients by R-package glmnet v4.1.1. Genes with coefficients in multivariate Cox model of zero were excluded. We designed a deep neuron network architecture by combining fully connected layers with Cox proportional hazards regression. A holdout 30% subset dataset in training cohort was used to evaluate and remainders were used to train the model by PyTorch v.1.8.1. Two external prospective datasets were used to validate the robustness of the prognostic model.

Statistical analysis

The univariate and multivariate Cox proportional hazards regression analyses were used to identify clinical features associated with survival. The survival rates and log-rank test of patients in high risk and low risk groups were estimated using Kaplan-Meier survival curve by R-package survival v3.2.7. We used R-package survminer v0.4.8 to generate survival plots. C-index is a goodness of fit measure for models that produce risk scores. To evaluate our risk model in survival analysis, Harrell's C-index was calculated for each validation dataset by R-package survival v3.2.7 (Harrell et al., 1982). HR, the ratio of event probabilities, was calculated by R-package survival v3.2.7. The time-dependent ROC curve was created to investigate whether the model could effectively predict survival using the R-package survivalROC v1.0.3. The bias-corrected and bootstrap CI were computed for c-indices and areas under the curves using 500 bootstrap replicates. A two-sided *P* value of less than 0.05 was considered significant, and the confidence level of CIs is 95%. The *P* values of other tests without specification were obtained via two-sided Student's *t*-test. All figures without specification were plotted with R-package ggplot2 v3.3.3.

Supplementary Figures

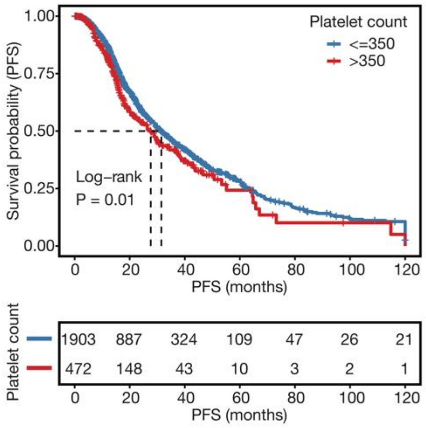

Figure S1. Thrombocytosis associates with ovarian cancer prognosis. Kaplan-Meier survival analysis showed that pre-treatment thrombocytosis was significantly associated with shortened progression-free survival in 2,404 patients with ovarian cancer.

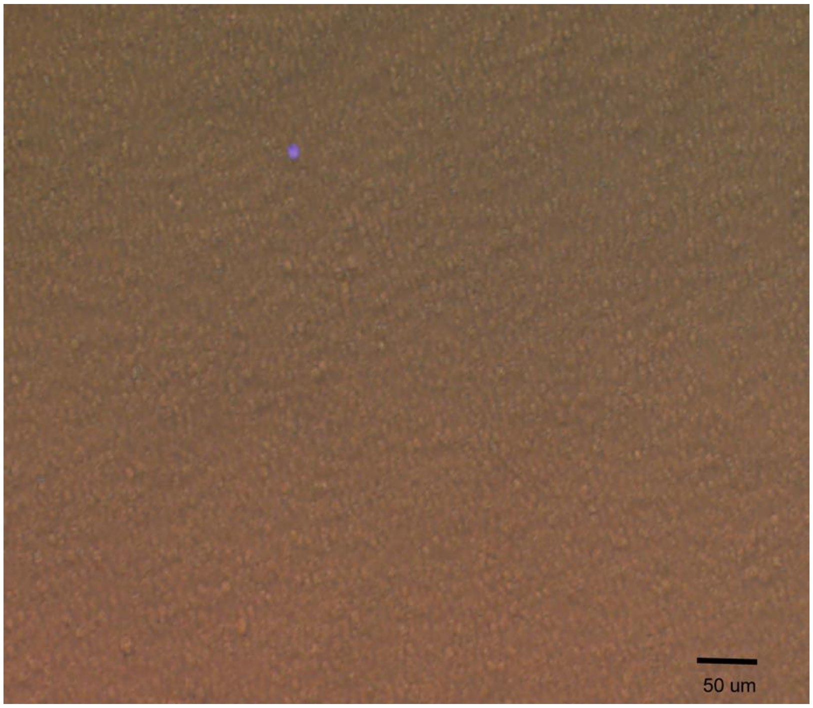

Figure S2. Representative graph of quality control for platelet purity. We adopted gradient centrifugation to isolate platelets based on previous literature (Best et al., 2015) and assessed platelet purity for all study samples by fixing platelets isolations in RNAlater in 3.7% paraformaldehyde and staining using DAPI. Total platelet and nucleated cell counts were determined by manual cell counting in 5 μL cell counting chambers on the fluorescence microscope. The results showed that 1 to 5 nucleated cells (stained with blue fluorescence, DAPI staining) were detected per 10 million platelets, which was consistent with the observations of previous literature (Best et al., 2015).

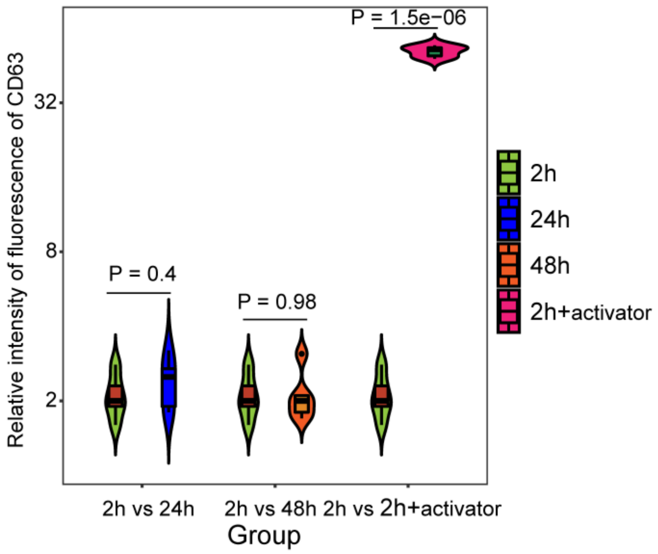

Figure S3. Quality control to assess platelet activation. We used flow cytometric analysis to measure platelet activation-dependent marker CD63 (Biolegend) to assess platelet activation during sample processing using a BD FACSCalibur flow cytometer. Four 6-mL EDTA-coated blood samples were collected from healthy donors, and platelet activation status was determined at 2 hours, 24 hours, and 48 hours. Platelets isolated at 2 hours using a standardized protocol that has been validated to induce minimal platelet activation (Best et al., 2015) were set as negative controls. Platelets activated by prothrombin (Sigma-Aldrich, 1 unit per mL) were positive controls. Platelet pellets after isolation were prefixed in 0.5% formaldehyde (Roth) for flow cytometric analysis. Relative activation and mean fluorescent intensity values were assessed. Stable levels of CD63 from samples at 24 hours and 48 hours suggested that platelets were not activated during blood collection and storage. Absence of platelet activation by standardized platelet isolation and storage protocol have also been validated by previous literature (Best et al., 2019)

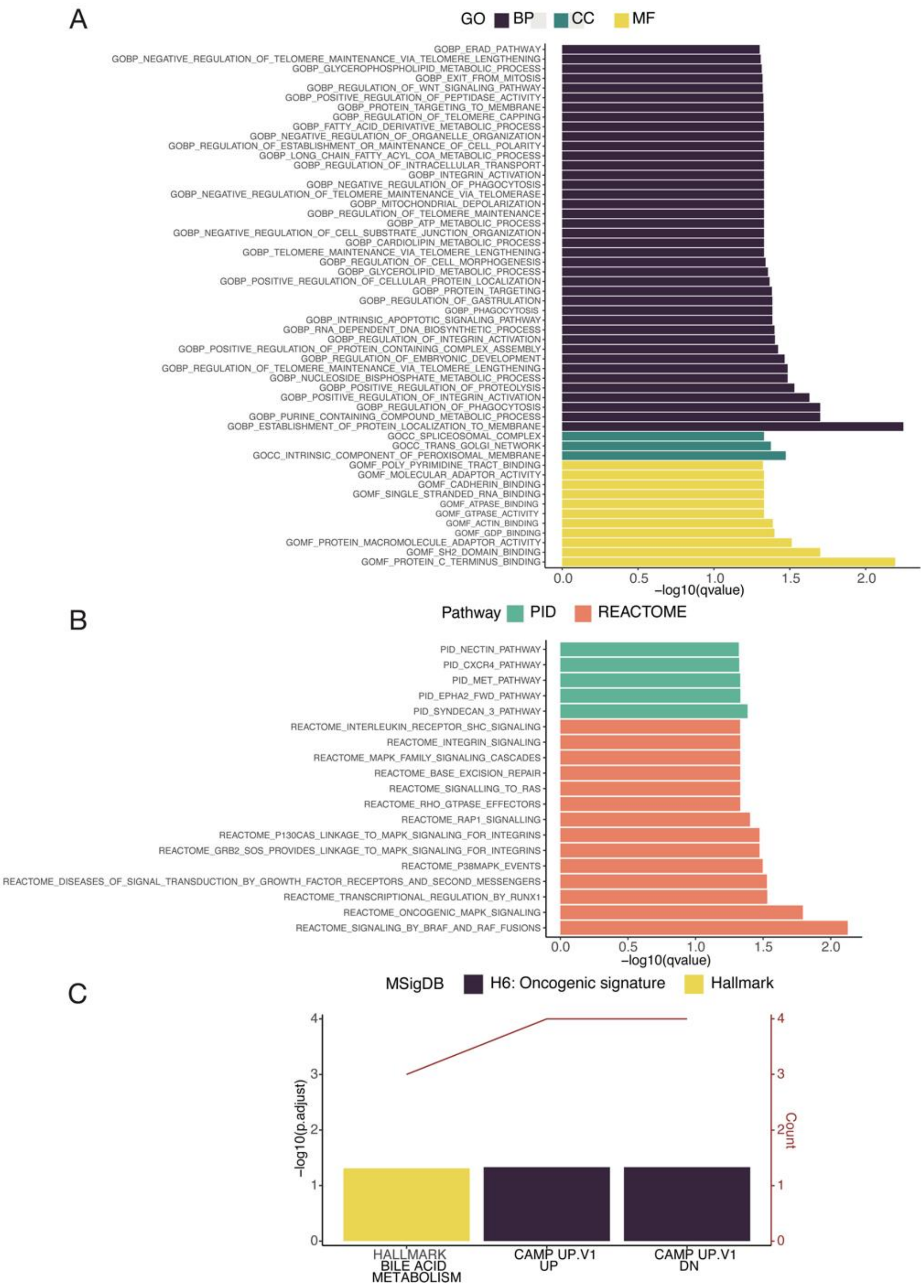

Figure S4. Enrichment analysis of the contributing genes of platelet RNA signature. The 100 genes were enriched based on gene ontology (GO) (Fig. S4A), REACTOME pathway database (Fig. S4B), Pathway Interaction Database (PID) (Fig. S4B), and Molecular Signatures Database cancer hallmarks (Fig. S4C), respectively.

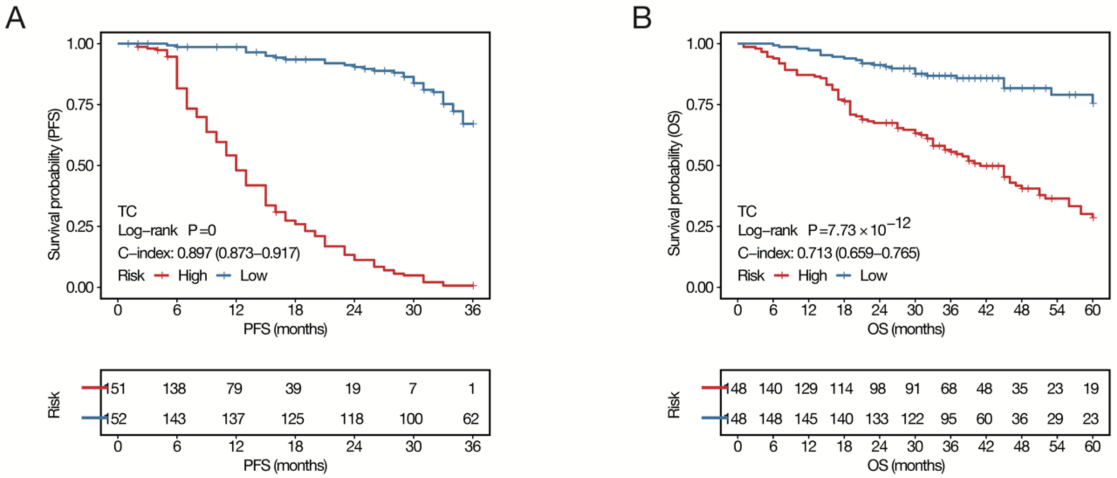

Figure S5. Kaplan-Meier analyses in training cohort. Kaplan-Meier plots for progression-free survival (A) and overall survival (B) analysis in the training cohort.

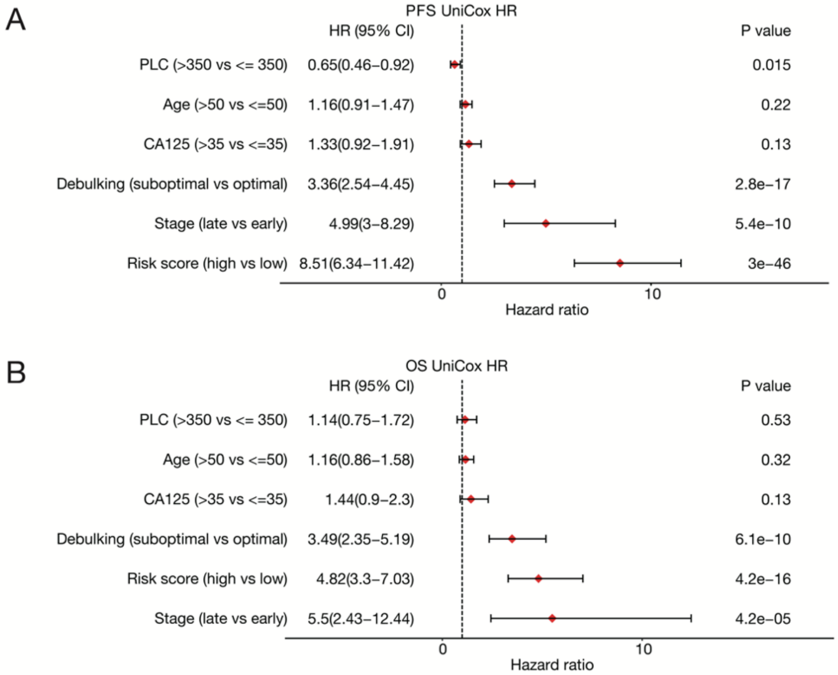

Figure S6. Univariate Cox regression analysis. Univariate Cox regression analysis to investigate the association between different variables (thrombocytosis, patient age, serum CA125 level, debulking status, disease stage, and DeepCox risk score) and PFS (A) or OS (B). The dashed vertical line in the forest plots represent an estimate of no effect. If the confidence intervals for individual factors overlap with the line, it demonstrates that at the given level of confidence, their effect sizes do not differ from no effect. PFS, progression-free survival. OS, overall survival. PLT, platelet count ($\times 10^9$ per L).

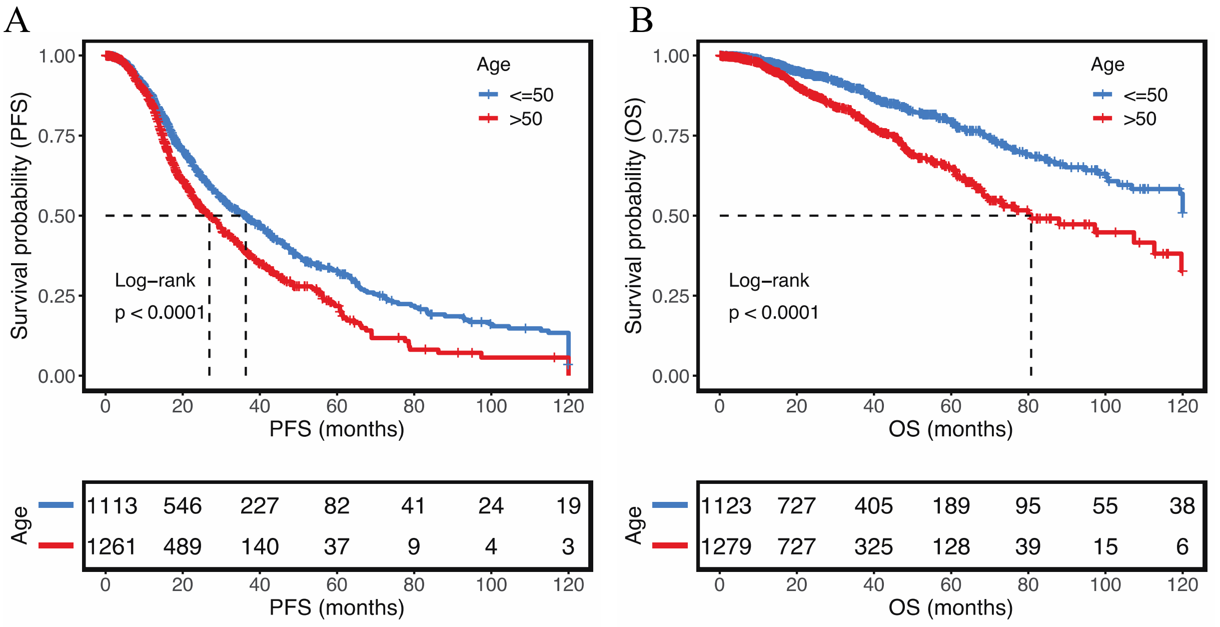

Figure S7. Advanced age associates with survival of ovarian cancer. Kaplan-Meier survival analysis shows that advanced age (>50) significantly associates with shortened progression-free survival (A) and overall survival (B) in 2,404 patients with ovarian cancer.

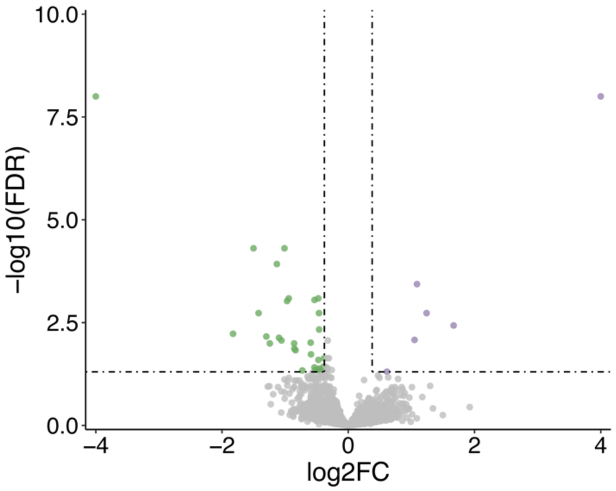

Figure S8. Differentially expressed genes of platelet transcriptome between chemotherapy responders and non-responders. A total of 36 differentially expressed genes were identified including six upregulated genes and 30 downregulated genes based on a fold change cut-off of 1.3 and FDR < 0.05.

References

Anders S, Pyl P, Huber, W (2015) HTSeq--a Python framework to work with high-throughput sequencing data. Bioinformatics 31:166–169

Best M, In 't Veld S, Sol N, Wurdinger T (2019) RNA sequencing and swarm intelligence-enhanced classification algorithm development for blood-based disease diagnostics using spliced blood platelet RNA. Nat Protoc 14:1206–1234

Best M, Sol N, In 't Veld S, Vancura A, Muller M, Niemeijer A, Fejes A, Tjon Kon Fat L, Huis In 't Veld A, Leurs C, et al (2017) Swarm Intelligence-Enhanced Detection of Non-Small-Cell Lung Cancer Using Tumor-Educated Platelets. Cancer Cell 32:238–252.e239

Best M, Sol N, Kooi I, Tannous J, Westerman B, Rustenburg F, Schellen P, Verschueren H, Post E, Koster J, et al (2015) RNA-Seq of Tumor-Educated Platelets Enables Blood-Based Pan-Cancer, Multiclass, and Molecular Pathway Cancer Diagnostics. Cancer Cell 28:666–676

Collins GS, Reitsma JB, Altman DG, Moons KG (2015) Transparent reporting of a multivariable prediction model for individual prognosis or diagnosis (TRIPOD): the TRIPOD statement. BMJ 350:g7594

Dobin A, Davis C, Schlesinger F, Drenkow J, Zaleski C, Jha S, Batut P, Chaisson M, Gingeras T (2013) STAR: ultrafast universal RNA-seq aligner. Bioinformatics 29:15–21.

Harrell FE Jr, Califf RM, Pryor DB, Lee KL, Rosati RA (1982) Evaluating the yield of medical tests. JAMA 247:2543–2546

Katzman JL, Shaham U, Cloninger A, Bates J, Jiang T, Kluger Y (2018) DeepSurv: personalized treatment recommender system using a Cox proportional hazards deep neural network. BMC Med Res Methodol 18:24

Kurman RJ, Carcangiu ML, Herrington CS, Young RH. WHO Classification of Tumours of Female Reproductive Organs [Internet]. France: IACR; 2014 [cited 2022 March 13]. Available from: https://publications.iarc.fr/Book-And-Report-Series/Who-Classification-Of-Tumours/WHO-Classification-Of-Tumours-Of-Female-Reproductive-Organs-2014

Leek, J (2014) svaseq: removing batch effects and other unwanted noise from sequencing data.

Nucleic Acids Res 42:e161

Love M, Huber W, Anders S (2014) Moderated estimation of fold change and dispersion for RNA-seq data with DESeq2. Genome Biol 15:550

Nagpal C, Yadlowsky S, Rostamzadeh N, Heller KA. Deep Cox Mixtures for Survival Regression. arXiv:210106536 [cs, stat] [Internet]. 2021 [cited 2022 March 13]; Available from: http://arxiv.org/abs/2101.06536

Prat J (2014) Staging classification for cancer of the ovary, fallopian tube, and peritoneum. Int J Gynecol Obstet 124:1–5

Ritchie M, Phipson B, Wu D, Hu Y, Law C, Shi W, Smyth G (2015) limma powers differential expression analyses for RNA-sequencing and microarray studies. Nucleic Acids Res 43:e47

Yates A, Achuthan P, Akanni W, Allen J, Allen J, Alvarez-Jarreta J, Amode M, Armean I, Azov A, Bennett R, *et al* (2020) Ensembl 2020. Nucleic Acids Res 48:D682–D688
