# Supplementary material for: Platelet RNA signature independently predicts ovarian cancer prognosis by deep learning neural network model
Source: Protein Cell. 2022 Nov 7;14(8):618–22. doi: 10.1093/procel/pwac053 (PMC10392027; doi:10.1093/procel/pwac053)
Supplement: pwac053_suppl_Supplementary_Table_S2 [file pwac053_suppl_supplementary_table_s2.pdf]

Table S2. Participant characteristics at baseline.

	Training cohort (*N*=303)	Validation cohort 1 (*N*=39)	Validation cohort 2 (*N*=83)
Age, years, median	51 (45–59)	52 (47–56)	52 (46–59)
Histology			
Serous	228	29	53
Mucinous	26	5	9
Endometrioid	20	2	4
Clear cell	15	1	0
Other	14	2	17
FIGO stage			
I	30	4	12
II	20	2	8
III	184	23	52
IV	69	10	11
CA125, mU/L			
< 35	35	6	13
≥ 35	268	33	70
Platelet count, $\times 10^9$/L, median	238 (188–328)	203 (155–360)	237 (163–300)
OS, months, median	37 (27–46)	33 (24–55)	25 (21–41)
PFS, months, median	20 (10–34)	23 (9–31)	15 (9–24)
Treatment			
Platinum-based chemotherapy	253	27	71
Platinum sensitive	181	18	54
Platinum resistant	72	9	17
PARP inhibitors	7	0	2
Anti-angiogenesis drugs	26	1	16

Data are n or median (IQR). *Abbreviations:* OS, overall survival. PFS, progression-free survival. PARP inhibitors, poly (ADP-ribose) polymerase inhibitors. IQR, interquartile range.
